# Supplementary material for: Farmer preference for macadamia varieties and constraints to production in Malawi
Source: PLoS One. 2024 Feb 23;19(2):e0293488. doi: 10.1371/journal.pone.0293488 (PMC10889898; doi:10.1371/journal.pone.0293488)
Supplement: S1 Table — (DOCX) [file pone.0293488.s001.docx]

**Farmer preference for macadamia varieties and constraints to production in Malawi**

Emmanuel Junior Zuza^1, #a, 2, #b, *^, Yoseph N. Araya^2^, Kadmiel Maseyk^2^, Shonil Bhagwat^3^, Rick L. Brandenburg^4^, Andrew Emmott^5^, Will Rawes^5^, Patrick Phiri^6^, Ken Mkengala^7^, Edwin Kenamu^8^

S1 Table: Characteristics of macadamia varieties cultivated in Malawi.

| Cultivar | Origin | OG | NG | Tree shape | Kernel nut size | Kernel weight (g) | Recovery (%) | Harvesting | Husk thickness | Susceptibility to TNB | Susceptibility to SGSB | Close planting |
| --- | --- | --- | --- | --- | --- | --- | --- | --- | --- | --- | --- | --- |
| HV A4 | Australia |  |  | Spreading | Large | 2.8–3.2 | 40–45 | Mid | Thin | High | High | Yes |
| HAES 246 | Hawai’i |  |  | Spreading | Large | 2.0–3.0 | 35–37 | Mid-late | Variable | Intermediate | Intermediate | No |
| HAES 333 | Hawai’i |  |  | Round | Small | 1.8–2.4 | 30–40 | Mid-late | Thick | Low | Low | No |
| HAES 344 | Hawai’i |  |  | Upright | Medium | 2.0–2.5 | 32–35 | Early | Variable | Intermediate | Intermediate | Yes |
| HAES 508 | Hawai’i |  |  | Spreading | Medium | 2.0–2.5 | 40–45 | Very late | Variable | Intermediate | Intermediate | No |
| HAES 660 | Hawai’i |  |  | Upright | Small | 1.8–2.3 | 33–39 | Very late | Thin | High | High | Yes |
| HAES 705 | Hawai’i |  |  | Spreading | Large | 2.3–2.8 | 34–35 | Very late | Thick | Low | Low | No |
| HAES 741 | Hawai’i |  |  | Upright | Medium | 2.0–2.5 | 32–37 | Early | Thin | High | Intermediate | Yes |
| HAES 772 | Hawai’i |  |  | Spreading | Medium | 2.0–2.8 | 26–29 | All year | Variable | Intermediate | Intermediate | Yes |
| HAES 781 | Hawai’i |  |  | Upright | Large | 2.6–3.0 | 34–38 | Very late | Thin | Intermediate | High | No |
| HAES 783 | Hawai’i |  |  | Spreading | Medium | 2.2–2.4 | 38–40 | Very late | Thick | Low | Low | No |
| HAES 788 | Hawai’i |  |  | Spreading | Medium | 2.2–2.4 | 38–40 | All year | Thin | High | Intermediate | No |
| HAES 791 | Hawai’i |  |  | Upright | Medium | 2.0–2.5 | 34–35 | All year | Variable | Intermediate | High | Yes |
| HAES 800 | Hawai’i |  |  | Spreading | Large | 2.0–3.0 | 35–40 | All year | Thick | Low | Low | Yes |
| HAES 814 | Hawai’i |  |  | Upright | Small | 1.8–2.4 | 37–39 | Mid-late | Thin | High | High | Yes |
| HAES 816 | Hawai’i |  |  | Upright | Large | 2.0–2.5 | 42–45 | Early | Thin | High | High | Yes |
| HAES 842 | Hawai’i |  |  | Upright | Medium | 2.0–2.5 | 35–39 | Early | Variable | Intermediate | Intermediate | Yes |
| HAES 849 | Hawai’i |  |  | Spreading | Small | 1.8–2.3 | 35–40 | Mid-late | Thin | High | High | No |
| Beaumont | Australia |  |  | Spreading | Medium | 2.0–2.5 | 33–39 | Mid | Thick | Low | Low | Yes |
| Daddow | Australia |  |  | Spreading | Medium | 2.0–2.4 | 37–40 | Mid-late | Thin | High | High | No |
| MCT1 | Australia |  |  | Spreading | Large | 2.0–3.0 | 38–40 | Mid-late | Variable | Intermediate | Intermediate | Yes |

Note: OG – Old generation cultivars and NG – New Generation cultivars.

: HV A – Hidden Valley Australia cultivars and HAES - Hawai’i Agricultural Experimental Station cultivars (Bell et al., 1996)
