# Supplementary material for: Farmer preference for macadamia varieties and constraints to production in Malawi
Source: PLoS One. 2024 Feb 23;19(2):e0293488. doi: 10.1371/journal.pone.0293488 (PMC10889898; doi:10.1371/journal.pone.0293488)
Supplement: Attachment — Submitted filename: Response to reviewers.docx [file pone.0293488.s003.docx]

Royal Agricultural University

Stroud Road

Cirencester

United Kingdom

GL7 6JR

[**https://rau.ac.uk/**](https://gateway.rau.ac.uk/?redirect=0)

**The Editor in Chief**

PLOs One Journal.

Dear Editor in Chief,

**RE: Farmer preference for macadamia varieties and constraints to production in Malawi**

I would like to express my gratitude on behalf of all the authors for the invaluable feedback provided by the Academic Editor and the Reviewers. We have carefully considered each comment and suggestion, and we believe that our revised manuscript now aligns with PLOS ONE's style requirements and addresses the concerns raised. Please find below our responses to each point raised:

**Responses to Academic Editor**

File naming and style requirements:

- This has been addressed, and the manuscript now adheres to PLOS ONE's style requirements, including proper file naming.

Inclusivity in global research questionnaire:

- The complete copy of PLOS' questionnaire on inclusivity in Global research has been included in the revised manuscript.

Role of funders:

- The role of funders in the study has been clearly stated in the new version of the manuscript.

Removal of funding-related text:

- All funding-related text has been removed from the manuscript as recommended.

Data accessibility:

- The questionnaire data has also been made available [here](https://figshare.com/articles/dataset/Farmer_preference_for_macadamia_varieties_and_constraints_to_production_in_Malawi_Raw_data/24866670) uploaded. Focus group discussion data, being sensitive, is available upon request.

Permission for figures:

- All data from [OpenTopography](https://portal.opentopography.org/datasets?minX=-3.1448364257812496&minY=51.1228121096031&maxX=-0.7992553710937501&maxY=52.25334270231613) is freely available and published under the CC BY 4.0 license.

**Responses to Reviewer #1:**

Summarizing key points:

- Quotes from focus group discussions have been retained to maintain the integrity of the study and inform policy. However, we have summarised them as suggested.
- Similar styles have been employed in transdisciplinary papers as well and hence the reason of us doing this.

Connectives and descriptives:

- Redundant connectives and verbose descriptives have been removed as recommended.

Unnecessary introductions:

- Unnecessary introductions to sections and sub-sections have been excluded in the revised manuscript.

Quotes from survey answers:

- Quotes from survey answers have been retained, considering the mixed methods approach used for data collection.
- The reason for this decision is because the qualitative data enriches the manuscript and provides an importance of including community knowledge in research which is currently being advocated for. Some examples of other papers that have done the same include:
  - <https://journals.plos.org/plosone/article?id=10.1371/journal.pone.0290877>
  - <https://journals.plos.org/plosone/article?id=10.1371/journal.pone.0272126>
  - <https://rmets.onlinelibrary.wiley.com/doi/pdf/10.1002/cli2.55>
  - <https://journals.plos.org/climate/article?id=10.1371/journal.pclm.0000082>
  - <https://journals.ametsoc.org/view/journals/wcas/14/2/WCAS-D-21-0075.1.xml>

**Responses to Reviewer #2**:

Relevance outside of Malawi:

- Policy recommendations have been included to emphasize the broader relevance beyond Malawi.
- For example, we advocate for informed breeding programs that align with farmer preferences to promote greater adoption of macadamia varieties (the reason for this is because majority of macadamia breeding programs do not consider smallholder farmer preferences rather commercial estates).

Clarification of macadamia prices:

- The statement regarding macadamia prices has been revised to provide clarity on the crop's returns.
- It now reads: Because the crop's returns are higher (~$14 to 15 kg-1) than other cash crops, the country's macadamia industry is rapidly expanding, with farms previously used for coffee, tea, and tobacco production being converted or diversified to macadamia production [10,11].

Explanation of symbol :

- The symbol "For all" has been explained for better clarity in the manuscript.
-
Note for all varieties in set with elements j, k, the farmer chooses a variety that gives them the most utility/profit when they compare j and k.

Correction of writing error:

- The writing error of requiresa has been changed to: requires a substantial variation in the dependent and independent outcomes, preferably from a large enough sample size
- The writing error regarding the dependent binary variable has been rectified.

Verification of cultivar names:

- The cultivar names have been verified, and the correct information has been incorporated into the manuscript.
- Note Keaau is an old name of HAES 660 base on the following publication ([here](https://era.daf.qld.gov.au/id/eprint/1964/14/mac-varieties.pdf)).

Amendment of varietal preferences in Mwanza cooperative:

- Preferences for varieties in Mwanza have been amended to accurately reflect the data i.e. HAES 741 and 800 are the most preferred (24%).

Clarification on "Active Farming Years":

- The explanation of considering 50 years as less active in farming in the context of Malawi has been provided.
- In Malawi once you are at the age of 50 and above you are considered less or not active in farming. The reason being the use of hand-held implements and at that age the amount of work you can do is limited and hence the expression.

Does this refer to Malawi? The sentence is a bit confusing:

- The sentence referring to Malawian macadamia processors has been rephrased for clarity.

Compared with areas with more seasonal growth patterns?

- Navigating this question proves challenging as our available data lacks support for the underlying assumption. Consequently, we opted to narrow our focus to examine the impact of topography on the flowering patterns of HAES 791 in comparison to HAES 660. The manuscript underscores that certain varieties exhibit extended flowering periods, while others undergo flowering for only 2 to 3 months, contingent upon the specific geographical area.

What does generations refer to here? Distinct periods of cultivar development?

- The answer is Yes. We have rectified this to avoid the confusion.

How does this pattern of variety selection compare with other places globally? For instance, 344 is a popular cultivar in Hawaii:

- The manuscript now includes information on how variety selection differs between smallholder and commercial farmers in various regions.
- It is essential to note that there are significant disparities in how variety selection is conducted between smallholder and commercial producers of macadamia. Among smallholders in Malawi and Kenya, individuals typically lack influence over the varieties they cultivate and are not actively engaged in breeding and introduction programs. They rely on recommendations from agronomists associated with commercial estates, often situated in different production regions. The high significance of our manuscript lies in emphasizing the crucial inclusion of smallholders in varietal breeding and introduction research.
- Regarding HAES 344, its popularity in Hawaii stems from its specialized breeding for the region. Notably, it is currently being replaced by newer varieties. Additionally, this particular variety is predominantly cultivated in Malawi and not in South Africa (RSA) and Kenya among commercial producers.

Bigger-picture contribution:

- The manuscript now explicitly states the lessons learned and the broader contributions to varietal breeding research, especially in areas with smallholder macadamia production.
- Our research holds relevance for various production areas and systems. It marks the initial study focusing on smallholder farmer macadamia varietal preferences. In regions where smallholder macadamia production is prevalent, crucial lessons have been gleaned. For instance, before introducing or breeding macadamia varieties, it is imperative to involve smallholders in the research process.
- An illustrative example is found in Mozambique, where smallholders, encouraged to use the Beaumont variety as a rootstock, encountered significant challenges due to unsuitable conditions. This highlights the potential pitfalls of adopting varieties without considering local conditions.
- Furthermore, while estate producers often use well-researched varieties tailored to specific regions, the diverse microclimates within a country, such as Malawi, necessitate caution. Our study reveals regional variations, such as HAES 791's longer flowering period in specific parts of the country, emphasizing the importance of localized research.
- These findings have broader applicability beyond Malawi, offering insights for macadamia cultivation in diverse regions worldwide. Additional sentences have been incorporated to underscore these points as recommended.

Citation software error:

- The citation software error regarding "du Toit" has been rectified.

We believe that these revisions address the concerns raised by the editor and reviewers comprehensively. We appreciate the time and effort invested in reviewing our manuscript, and we are confident that the changes made enhance the overall quality of the submission.

Thank you for your consideration.

Sincerely,

Emmanuel Junior Zuza (PhD).
